# Ipilimumab and Its Derived EGFR Aptamer-Based Conjugate Induce Efficient NK Cell Activation against Cancer Cells

**DOI:** 10.3390/cancers12020331

**Published:** 2020-02-01

**Authors:** Margherita Passariello, Simona Camorani, Cinzia Vetrei, Stefania Ricci, Laura Cerchia, Claudia De Lorenzo

**Affiliations:** 1 Department of Molecular Medicine and Medical Biotechnology, University of Naples “Federico II”, Via Pansini 5, 80131 Naples, Italy; margherita.passariello@unina.it (M.P.); cinzia.vetrei@unina.it (C.V.); stefaniaricci1992@libero.it (S.R.); 2 Ceinge – Biotecnologie Avanzate s.c. a.r.l., via Gaetano Salvatore 486, 80145 Naples, Italy; 3 Institute of Experimental Endocrinology and Oncology “Gaetano Salvatore” (IEOS), CNR, Via S. Pansini 5, 80131 Naples, Italy

**Keywords:** EGFR, CTLA-4, bispecific, natural killer cells, ipilimumab, aptamer, cancer therapy

## Abstract

The immune checkpoint CTLA-4 (cytotoxic T-lymphocyte-antigen 4), which inhibits the co-stimulatory CD28 signal on T cells, has been recently found expressed on other cell populations, such as tumor and natural killer (NK) cells. We tested for the first time the effects of ipilimumab, the human anti-CTLA4 mAb in clinical use, on these cells and found that it inhibits the growth of tumor cells expressing CTLA-4 also in the absence of lymphocytes, and efficiently activates NK cells, thus suggesting an important unexplored role of NK cells in ipilimumab-modulated immune responses. Interestingly, the epidermal growth factor receptor (EGFR) has been shown to play a key role in tumor cell escape from immune surveillance, and in cytotoxic T lymphocyte inhibition. Thus, we tested combinatorial treatments of ipilimumab with an anti-EGFR aptamer endowed with anti-tumor activity, and constructed for the first time a novel bispecific immunoconjugate, made up of these two compounds. The novel immunoconjugate binds to the target cells, induces the activation of lymphocytes, including NK cells, and inhibits the growth of tumor target cells more efficiently than the parental compounds, by strongly enhancing the cytotoxic activity of both human peripheral blood mononuclear cells and NK cells against tumor cells.

## 1. Introduction

Cancer immunotherapy approaches based on the use of antitumor or immunomodulatory mAbs [1] have been recently improved by the last clinical success of the novel human antibodies approved by the Food and Drug Administration (FDA), such as ipilimumab and nivolumab [2,3]. In particular ipilimumab recognizes the cytotoxic T lymphocyte-antigen 4 (CTLA-4), also known as CD152, which plays a crucial role in T cell inhibition [4]. Indeed, CTLA-4 is upregulated on activated T cells [5] where competes with T cell-associated CD28 receptor by binding to B7-1/B7-2 ligands [6], expressed on antigen-presenting cells [7,8], thus blocking the co-stimulatory CD28 signal necessary for full T-cell activation. 

CTLA-4 is also constitutively expressed on T regulatory cells (T reg), which can impair immune response by mediating the removal of B7-1 and B7-2 through trans-endocytosis, thus reducing their stimulatory function [9,10]. In addition, the CTLA-4 cytoplasmatic tail contains a tyrosine motif, which can be phosphorylated, thus creating harbors for SH2 domain of phosphatases and for other intracellular proteins such as the p85 subunit of phosphatidylinositol 3 kinase (PI3K) [11]. Recently, it has been reported that effector and T reg cells are not the only populations of the immune system exhibiting this antigen, as also natural killer (NK) cells display CTLA-4 on their surface, whose role has not yet been fully clarified [12]. Furthermore, recent studies reported that this protein is also expressed on non-lymphoid cells including leukemia and solid tumor cells [13,14,15,16] and although its role in tumor cells has not yet been fully elucidated, some reports indicate that it can affect tumor cell survival [15,17]. 

The recognition of the important role of CTLA-4 led to the development of inhibitory antibodies blocking its function, as an attractive strategy for cancer treatment. After preliminary positive outcomes in mice models, the fully human anti-CTLA-4 mAb, ipilimumab, has been developed from Allison’s research group in 1996 [18]. Ipilimumab was approved by the U.S. FDA in 2011 for the treatment of metastatic melanoma [2,19], and several clinical trials have been started for other solid tumors, such as non-small-cell lung cancer (NSCLC), and renal cell and prostate carcinomas [20]. Nevertheless, the monotherapy approach seemed to be not effective in the case of poor immunogenic tumors, thus inducing studies and trials focused on the combination of ipilimumab with other anti-tumor drugs, such as IL-2, peptide vaccine, chemotherapy drug decarbazine [21,22,23], or with other immune checkpoint inhibitors. Among them, the anti-PD-1 mAb nivolumab, in combination with ipilimumab, has shown two-year overall survival (OS) rates of 40% in patients affected by NSCLC [24]. Moreover, in 2019 ipilimumab has been approved by European Medicines Agency for the treatment of renal cell carcinoma and by FDA for the treatment of metastatic colorectal cancer in combination with nivolumab [25,26]. 

Further, the oncogenic behavior of epidermal growth factor receptor (EGFR), combined to its recently emerged role as important modulator of immune response [27], prompted the evaluation of ipilimumab plus EGFR-tyrosine kinase inhibitor (TKI) erlotinib in a clinical trial in patients with NSCLC [28,29]. Unfortunately, despite the significant outcome in terms of progression-free survival and OS, the trial was prematurely closed because of the severe side effects of erlotinib [28,29]. Nonetheless, this combinatorial strategy deserves to be further investigated, by overcoming the side effects related to erlotinib, by using a more specific anti-EGFR drug. Remarkably, activation of EGFR not only promotes tumor cell escape from immune surveillance, but it also inhibits the activity of cytotoxic T lymphocytes (CTLs) through different mechanisms such as the increased lactate excretion, the activation of T regs and the decrease of MHC I and II expression [30]. 

In order to study the efficacy of new combinatorial treatments involving anti-CTLA-4 and anti-EGFR drugs, we decided to test ipilimumab on a panel of tumor cells with other anti-EGFR specific compounds, such as aptamers [31]. Aptamers are single-stranded oligonucleotides, able to fold into a three-dimensional structure, by which they interact at high affinity and specificity with their targets. Their unique chemical and biological characteristics, i.e., small size, high stability, and lack of immunogenicity, can be exploited in cancer therapeutics, used as inhibitors of their specific targets, or for cell-targeted delivery of secondary therapeutic moieties [32,33]. In this regard, we used the validated anti-EGFR CL4 nuclease-resistant RNA-aptamer, generated in our laboratory, which was previously found to inhibit the derived tumor growth of NSCLC [33], glioblastoma [34,35], and triple-negative breast cancer (TNBC) [36,37]. 

Herein, we tested the combinatorial treatment of ipilimumab mAb plus CL4 aptamer on tumor cells and on immune cells, such as NK cells, since the latter are often infiltrated in some solid tumors [38] of patients responding to the immunotherapy, and we wanted to further evaluate their role in anti-tumor immune response as well as the possibility to potentiate their activity. 

On the basis of the promising results obtained with combined treatments, we also developed a novel bispecific chimeric molecule by chemically linking [39] the anti-EGFR aptamer to ipilimumab mAb. Here we characterized the biological activity of this novel immunoconjugate by testing its effects on tumor cells, on immune cells and on co-cultures of tumor and immune cells. 

## 2. Results

### 2.1. Effects of Ipilimumab and CL4 Aptamer on Tumor and Normal-Like Cells

**Figure 1:**
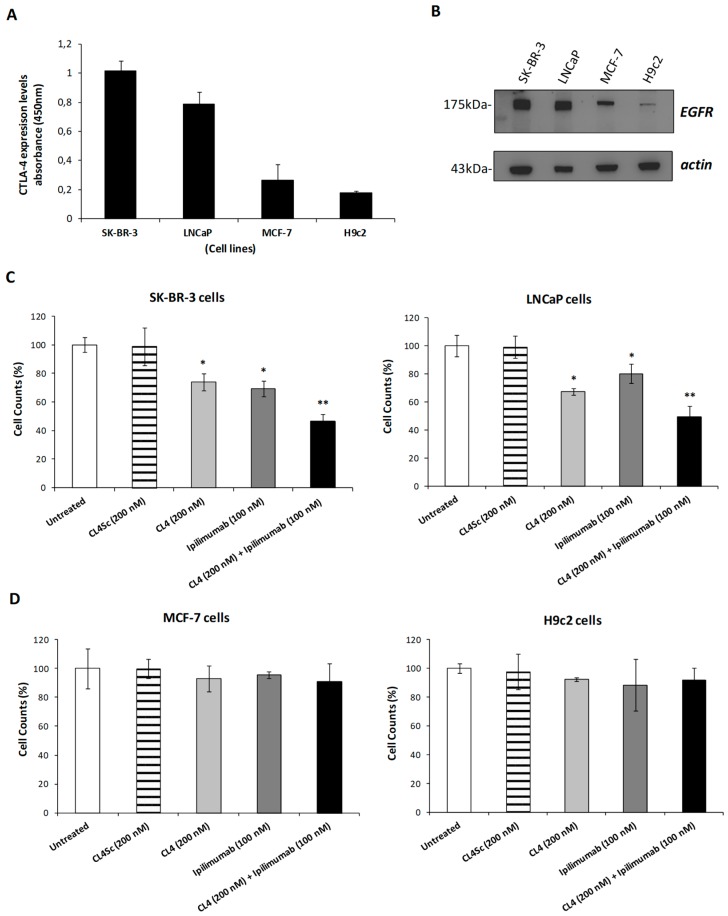
Effects of CL4 aptamer and ipilimumab mAb on tumor cells expressing different levels of CTLA-4 and EGFR. (**A**) Cell ELISA assays on SK-BR-3, LNCaP, MCF-7 tumor cells, and H9c2 cardiomyoblasts by using the anti-CTLA-4 mAb ipilimumab to measure the cell surface CTLA-4 expression level. (**B**) Western blotting analysis with the commercial anti-EGFR mAb of extracts from SK-BR-3, LNCaP, MCF-7, and H9c2 cells. The intensity of the bands was normalized to actin by calculating the ratio of EGFR/actin signal intensities for each cell extract that was found to be 4.5 for SK-BR-3, 5.2 for LNCaP, 0.7 for MCF-7, and 0.07 for H9C2, respectively. (**C**,**D**) CL4 aptamer (light grey bars), ipilimumab mAb (dark grey bars), or their combination (black bars) were incubated at the indicated concentrations for 72 h with SK-BR-3 and LNCaP tumor cells expressing both EGFR and CTLA-4 at high levels, or with MCF-7 and H9c2 cells expressing low levels of the antigens. Untreated cells (white bars) or CL4Sc aptamer (striped bars) were used as negative controls. Error bars depict means ± SD. p-values for the indicated treatments relative to untreated cells are: ** *p* < 0.01; * *p* < 0.05.

Recent reports [17] have evidenced the unexpected expression of CTLA-4 on tumor cells, thus, we investigated the effects of the human anti-CTLA-4 mAb ipilimumab [22] on a panel of cell lines expressing different levels of this antigen to verify whether it has also a direct anti-tumor cell activity independent from the immune response. To this aim, we firstly examined the expression of CTLA-4 on the surface of different cell lines by cell enzyme-linked immunosorbent assays (ELISA) (Figure 1A). CTLA-4 expression was detectable at high levels on the surface of breast SK-BR-3 and prostate LNCaP cancer cells, at very low levels on MCF-7 tumor cells and H9c2 cardiomyoblasts, used as control non-neoplastic cells. To accurately quantify the expression of CTLA-4 on these two cell populations, a calibration curve obtained with the purified protein used at increasing concentrations in a parallel ELISA assay was used. By this method a value for CTLA-4 expression of about 50 and 40 ng/million cells was determined for SK-BR-3 and LNCaP cells, respectively.

In parallel, EGFR expression on these cell lines was analyzed by western blotting with a commercial anti-EGFR mAb (see Figure 1B). Interestingly, CTLA-4-positive SK-BR-3 and LNCaP cancer cells showed higher levels of EGFR [40,41] than those detected on cells expressing low levels of CTLA-4, such as tumor MCF-7 cells or H9c2 cardiomyoblasts.

To investigate on the role of CTLA-4 in tumor cells, we firstly tested the effects of ipilimumab on tumor cell growth when used in single treatment (Figure 1C,D). The antibody reduced the growth by 30% in SK-BR-3 and by 20% inLNCaP cells when incubated at a concentration of 100 nM for 72 h, suggesting that it directly inhibits the growth of CTLA4-positive tumor cells also independently from the immune system. In parallel, we tested the effects of the anti-EGFR CL4 aptamer [33] on these tumor cells and, according to our previous findings [39], we observed a significant inhibition of tumor cell growth when used at the dose of 200 nM for 72 h, whereas no effect was observed with a scrambled aptamer (CL4Sc) used as a negative control. As expected, both the antibody and the aptamer showed no significant effects on MCF-7 tumor cells and non-neoplastic cardiomyoblasts expressing very low levels of the two antigens and, thus, used as negative controls. On the basis of these results, we evaluated the effects of combinatorial treatments of ipilimumab with the anti-EGFR CL4 aptamer (Figure 1C,D). The combination of the two drugs reduced the cell growth of the double antigen-positive tumor cells (50%–60% inhibition), more efficiently than single-agent treatments, whereas no significant effects were observed on the cell lines used as negative controls, thus confirming the specificity of these drugs for their targets. In order to clarify whether the marked inhibition of tumor cell growth observed with the combinatorial treatment was due to a more potent effect on the extracellular-signal regulated kinase 1/2 (ERK1/2) pathway downstream EGFR, we analyzed the extracts of treated cells with a commercial anti-pERK antibody. As shown in the Figure S2, the combinatorial treatment strongly inhibited the phosphorylation of ERK, thus confirming that this combined treatment acts by inhibiting cell proliferation in line with previous reports indicating that inhibition of EGFR and ICs counteracts tumor cell growth [33,35,42].

### 2.2. Construction of a Novel anti-CTLA4-EGFR Immunoconjugate

**Figure 2:**
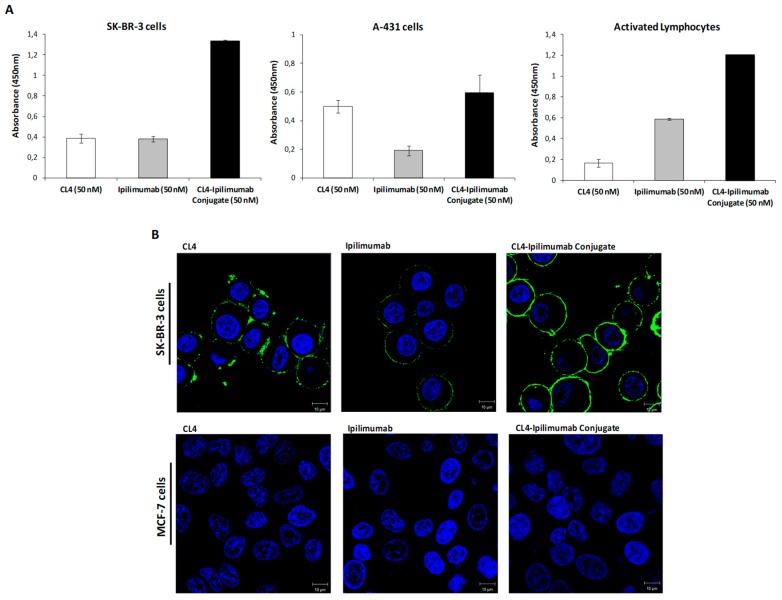
Binding of CL4-ipilimumab conjugate to tumor cells and lymphocytes. (**A**) Cell ELISA assays on SK-BR-3, A-431 cells or activated lymphocytes of CL4-ipilimumab conjugate (black bars), unconjugated CL4 aptamer (white bars), or ipilimumab mAb (grey bars), used as controls. Error bars depict means ± SD. (**B**) Representative confocal microscopy images of SK-BR-3 and MCF-7 cells treated with the indicated compounds. CL4, ipilimumab or CL4-ipilimumab conjugate are visualized in green. Nuclei are visualized in blue. Magnification 63×, 1.0× digital zoom. Scale bar = 10 μm.

On the basis of these promising results, and considering the impact of CTLA-4 and EGFR not only on tumor cell signaling pathways but also on the immune system [27], we decided to construct a novel immunoconjugate by chemically linking the Fc region of ipilimumab mAb with the amino-terminated CL4 aptamer, as we previously reported for other immunoconjugates [39]. The strategy used, based on the chemical modification of both the antibody and oligonucleotide [43], allowed the stable conjugation of the aldehyde-modified RNA aptamer with the hydrazinonicotinamide-incorporated antibody. The novel immunoconjugate, named CL4-ipilimumab, was firstly tested by cell ELISA assays on both tumor cells and lymphocytes for comparing its binding ability to that of the unconjugated parental moieties. As shown in Figure 2, the immunoconjugate, tested at the concentration of 50 nM, retains the binding ability of both the parental aptamer and antibody for their targets expressed on the surface of A-431 (EGFR-positive) tumor cells and activated lymphocytes (CTLA-4-positive) [5], respectively, but it also acquires a much higher avidity for double antigen-positive SK-BR-3 tumor cells. These results indicate that the linkage of the aptamer to the antibody does not affect the biological functions of the two compounds; on the contrary it provides a higher binding efficiency to the target cells. The specific binding of the CL4-ipilimumab conjugate to the surface of SK-BR-3 cells was further confirmed by confocal imaging. As shown (Figure 2B), the conjugate-associated signal on the cell surface appeared of higher intensity with respect to those obtained with the single parental aptamer or antibody, whereas, as expected, no signals were observed on negative control MCF-7 cells (Figure 2B).

### 2.3. Biological Activity of CL4-Ipilimumab

**Figure 3:**
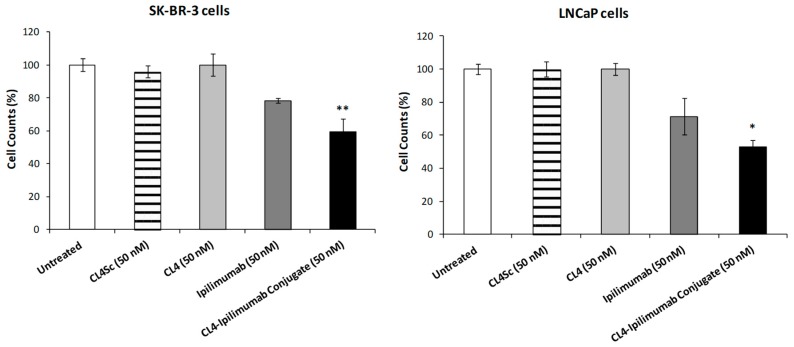
Effects of CL4-ipilimumab conjugate on tumor cell growth. Cell growth inhibition assays on SK-BR-3 and LNCaP cells, treated with CL4 aptamer (light grey bars), ipilimumab mAb (dark grey bars), or CL4-ipilimumab conjugate (black bars) for 72 h. Untreated cells (white bars) or cells treated with CL4Sc (striped bars) were used as negative controls. Error bars depict means ± SD. p-values for the indicated immunoconjugate relative to ipilimumab treated cells are: ** *p* < 0.01; * *p* < 0.05.

As a next step, we analyzed the effects of CL4-ipilimumab on tumor cell growth. To this aim, the double antigen-positive SK-BR-3 and LNCaP cells were treated for 72 h with CL4-ipilimumab, parental CL4 aptamer, or ipilimumab mAb used at a concentration of 50 nM, and cell growth was measured by counting trypan blue-excluding cells. As shown in Figure 3, the CL4 aptamer, when used as single agent at this lower concentration with respect to the one previous tested (200 nM), did not show significant effects and ipilimumab only slightly inhibited the growth of these tumor cells by reducing the cell counts by about 20%. The immunoconjugate instead showed an effect more potent than that of the single parental moieties by reaching about 50% of growth inhibition of LNCaP cells, thus indicating that the linkage of the two compounds provides the chimeric construct with enhanced inhibitory effects in line with the results of the combined treatment shown in Figure 1.

**Figure 4:**
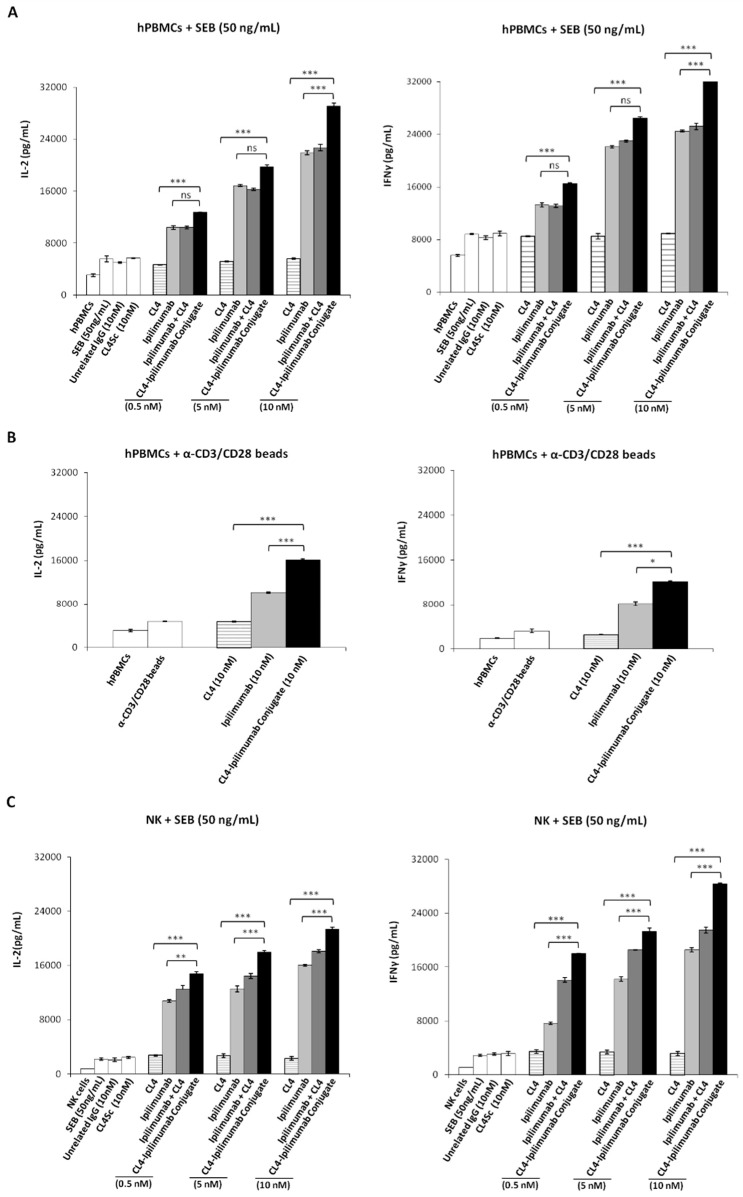
Effects of CL4-ipilimumab conjugate on cytokines secretion by different populations of hPBMCs. Levels of IL-2 and IFN-γ secreted from hPBMCs stimulated with SEB (**A**) or CD3^+^ T cells (PBMCs activated with α-CD3/CD28 beads) (**B**), or isolated NK cells treated wih SEB (**C**), in the absence or in the presence of CL4-ipilimumab conjugate (black bars), unconjugated CL4 (striped bars), ipilimumab (light grey bars), or their combination (dark grey bars) at the indicated concentrations for 66 h. Untreated or stimulated hPBMCs in the absence or in the presence of CL4Sc or unrelated IgG mAb were used as negative controls. Error bars depict means ± SD. *p*-values for the indicated compounds are: *** *p* ≤ 0,001; ** *p* < 0.01; * *p* < 0.05.

Considering the role of CTLA-4 in potentiating immune responses against cancer cells, and the new evidences suggesting the role of EGFR not only in the survival of tumor cells but also in the inhibition of immune system [27], we investigated the effects of the immunoconjugate also on the immune cells. Thus, we firstly measured the ability of the immunoconjugate to induce the proliferation and the activation of lymphocytes by measuring IL-2 and IFNγ cytokines secretion by unfractionated human peripheral blood lymphocytes (hPBMCs). To this aim, we stimulated the lymphocytes with Staphylococcal enterotoxin B (SEB) for 66 h, in the absence or in the presence of the CL4-ipilimumab conjugate, CL4 aptamer, ipilimumab mAb or their combination used at the concentration range of 0.5–10 nM. As shown in Figure 4A, CL4-ipilimumab activated the lymphocytes more efficiently than the parental mAb used alone or in combination with CL4 aptamer, thus supporting the hypothesis that the covalent linkage between the two moieties is beneficial also for a stronger activation of immune cells.

Since it has been recently reported that CTLA-4 antigen can be expressed not only by CD3^+^ T cells but also by subpopulations of NK cells in patients with advanced cancers [38], we decided to further investigate on the effects of the immunoconjugate on the two different lymphocytes populations, CD3^+^ T cells and NK cells, used separately in distinct assays. To this aim, the hPBMCs were treated with anti-CD3/anti-CD28 beads for 48 h to specifically activate the CD3^+^ T cells in the presence of the immunoconjugate or its parental compounds used as single agents (Figure 4B). In parallel, we tested the biological efficacy of the immunoconjugate, or the single parental compounds, on a fraction of lymphocytes population enriched in NK cells, isolated from hPBMCs as described in the Materials and Methods. Once isolated, the cells were stimulated with SEB for 66 h [44] in the absence or in the presence of the immunoconjugate (0.5–10 nM) or its parental compounds, used either as single agents or in combinatorial treatments (Figure 4C).

**Figure 5:**
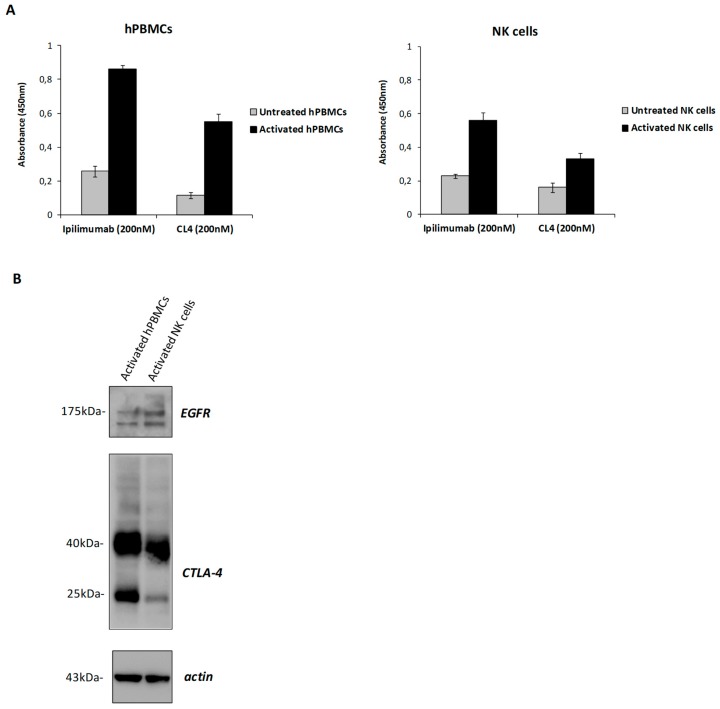
Expression level of EGFR and CTLA-4 on untreated or activated hPBMCs and NK cells. (**A**) Cell ELISA assays with ipilimumab and CL4 (200 nM) on hPBMCs and NK cells, untreated (grey bars) or activated (black bars) with α-CD3/CD28 beads or SEB, respectively. Error bars depict means ± SD. (**B**) Western blotting with commercial anti-CTLA-4 and anti-EGFR antibodies of cell extracts from activated hPBMCs or NK cells. The intensity of the bands was normalized to actin.

The results show that CL4-ipilimumab activated both the NK and CD3^+^ T cell populations more efficiently than the parental mAb used alone or in combination with CL4 aptamer, by inducing a more efficient secretion of both IL-2 and IFNγ cytokines. Furthermore, its effects and those of ipilimumab on NK cells are strong, suggesting, unexpectedly, that these lymphocytes are indeed important actors of the immune cell responses modulated by ipilimumab and CL4-ipilimumab conjugate. Our hypothesis is also confirmed by the increased expression of both CTLA-4 and EGFR on the surface of isolated NK cells upon their stimulation with SEB, measured by cell ELISA assays with ipilimumab and CL4 on both untreated or activated hPBMCs and NK cells, as shown in Figure 5. To confirm the expression of CTLA-4 and EGFR on NK cells by using an independent method we analyzed the cell extracts of activated hPBMCs and NK cells by Western blotting analysis with commercial anti-CTLA-4 and anti-EGFR antibodies. As shown in Figure 5B, the proteins of the expected molecular weight for the dimeric CTLA-4 (40–50 kDa) and EGFR (175 kDa) are clearly visible in both of the samples. The intensity of the band corresponding to CTLA-4 is about two-fold lower in NK cells than that observed in unfractionated hPBMCs containing CTLA-4-positive T cells, used as a positive control. Additional bands corresponding to the monomeric form of CTLA-4 (25 kDa) and to a truncated form of EGFR are also detectable. To accurately quantify the expression of CTLA-4 on these two cell populations, a calibration curve obtained with the purified protein used at increasing concentrations in a parallel ELISA assay was used. By this method a value for CTLA-4 expression of about 20–40 and 10–20 ng/million cells was determined for hPBMCs and NK cells, respectively.

**Figure 6:**
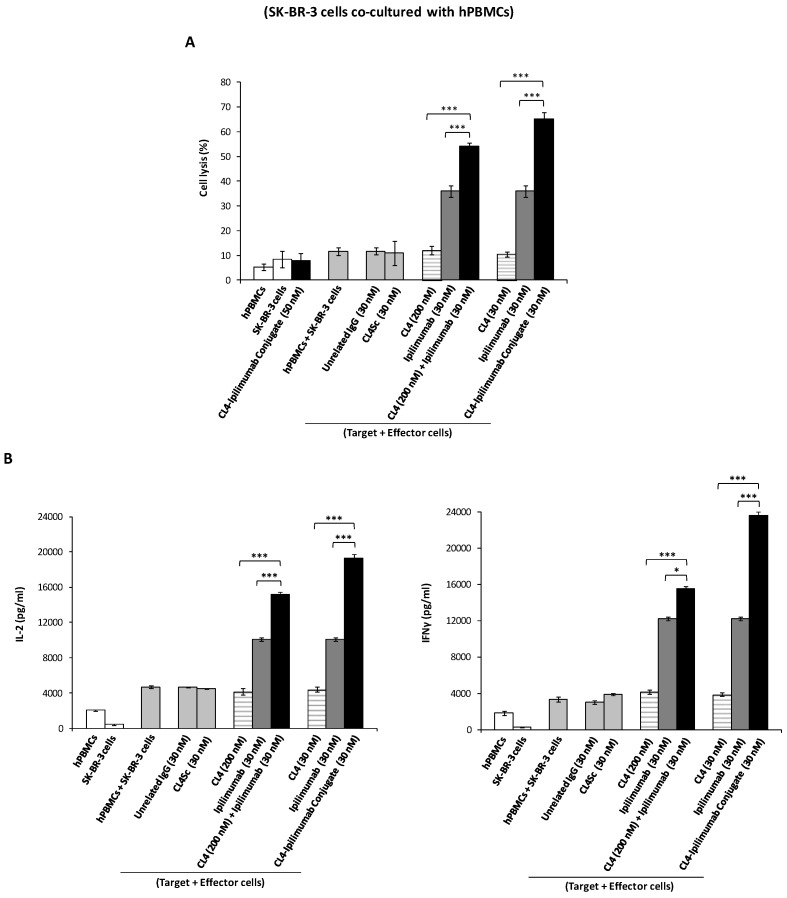
Cytotoxic effects of CL4-ipilimumab conjugate on SK-BR-3 tumor cells co-cultured with hPBMCs. (**A**) SK-BR-3 cells were co-cultured with hPBMCs (Effector:Target cells ratio 3:1) and treated for 24 h with CL4 aptamer (striped bars), ipilimumab mAb (grey bars), their combination or CL4-ipilimumab conjugate (black bars) at the indicated concentrations SK-BR-3 cells separately treated with the immunoconjugate, the scrambled aptamer, or with hPBMCs were included as controls. Cell lysis was measured as described in Materials and Methods. (**B**) IL-2 and IFN-γ secretion levels were measured by ELISA on supernatants of cells treated, as indicated. Error bars depict means ± SD. *p*-values for the indicated treatments are: *** *p* ≤ 0.001; * *p* < 0.05.

**Figure 7:**
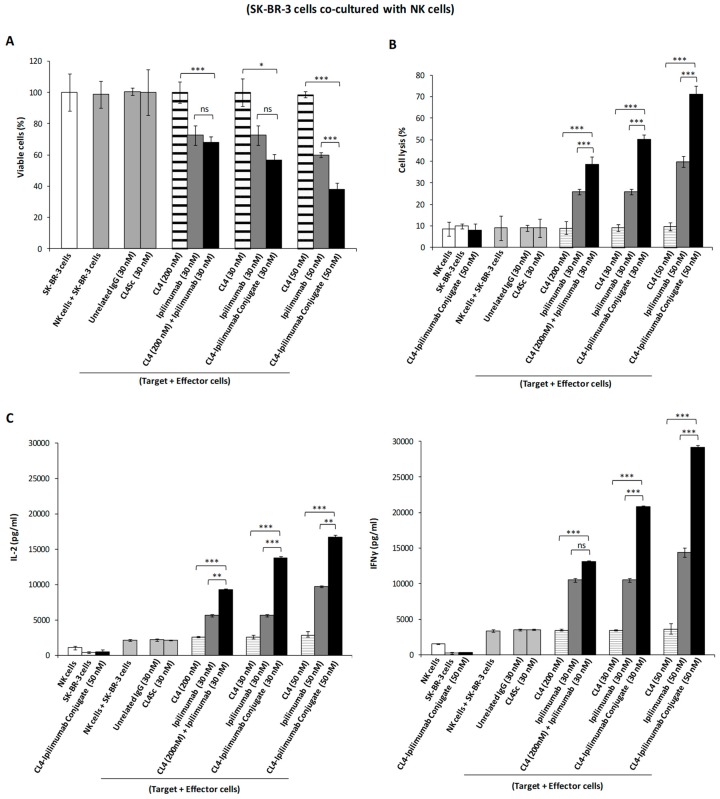
Cytotoxic effects of CL4-ipilimumab conjugate on SK-BR-3 tumor cells co-cultured with NK cells. (**A**) SK-BR-3 cells were co-cultured with NK cells (effector:target cells ratio: 3:1) and treated for 24 h with CL4 aptamer (striped bars), ipilimumab mAb (grey bars), their combination, or CL4-ipilimumab conjugate (black bars), as indicated. SK-BR-3 cells separately treated with the immunoconjugate, the scrambled aptamer, or with hPBMCs were included as controls. The percentage of viable cells is expressed with respect to untreated cells. (**B**) The release of lactate dehydrogenase was tested for measuring % cell lysis as described in Materials and Methods. (**C**) The levels of secreted IL-2 and IFN-γ were measured by ELISA on supernatants of cells treated, as indicated. Error bars depict means ± SD. *p*-values for the indicated treatments are: *** *p* ≤ 0.001; ** *p* < 0.01; * *p* < 0.05.

Consequently, we decided to further investigate these novel interesting effects of ipilimumab and its derived immunoconjugate also on co-cultures of tumor cells with either hPBMCs or NK cells, in order to verify whether the anti-tumor activity is enhanced by their combined effects on both tumor cells and immune cells in a microenvironment similar to that of in vivo solid tumors. To this aim, SK-BR-3 cells were co-cultured with hPBMCs or its fraction enriched in NK cells (effector:target ratio: 3:1) and treated for 24 h in the absence or in the presence of the immunoconjugate, its parental compounds used either as single agents or in combinatorial treatments at the same concentrations. As shown in Figure 6 and Figure 7, the immunoconjugate induced the death of tumor cells in a dose-dependent fashion and more efficiently than both the parental moieties, by significantly increasing the secretion of IL-2 and IFNγ cytokines by both hPBMCs and NK populations, thus leading to increased cell lysis with a higher lactate dehydrogenase (LDH) release by tumor cells. In order to confirm that CL4-ipilimumab conjugate induces the proliferation of NK cells, we measured the number of lymphocytes before and after the co-culture in the absence or in the presence of the indicated compounds. We found that ipilimumab increased the proliferation of 30%, whereas the conjugate induced about 2-fold increase thus significantly enhancing the effect of ipilimumab. 

Noteworthy, in these conditions the effects also exerted by the antibody and its derived immunoconjugate on the two co-cultures of tumor cells and hPBMCs or NK cells are comparable, thus suggesting that NK cells are responsible for most of the immune responses mediated by hPBMCs and modulated by ipilimumab and its derived immunoconjugate. Altogether these results confirm that CTLA-4 is indeed expressed on tumor cells and on NK cells and it plays a critical role in NK cell anti-tumor activity. Hence, taking this into consideration, the use of this novel immunoconjugate could improve the anti-tumor effects of ipilimumab by activating different lymphocytes populations with a higher potency than ipilimumab and by cross-linking tumor cells with immune cells.

## 3. Discussion

The first in class immunomodulatory mAb ipilimumab, currently in clinical use for advanced melanoma and undergoing a large number of clinical trials for the therapy of lung and prostate cancers, has been used in this work for the following two purposes: the first one was to confirm the expression and the role of CTLA-4 in tumor cells and NK cells as, recently, some authors have reported the unexpected expression of this receptor on these cell populations [12,14,17,38], even though its role in this context has not yet been accurately investigated and only little attention has been paid. The second aim was the use of this antibody for the production of a novel bispecific aptamer–antibody conjugate endowed with a potential increased anti-tumor efficacy.

CTLA-4 has been considered for a long time an exclusive immune receptor of T cells involved in their inhibition by switching off the downstream signaling of the T-cell co-stimulatory protein, CD28, as CTLA-4 binds to CD80 and CD86 with greater affinity and avidity than CD28 thus competing with it for its ligands [6]. However, its expression on other cell populations, such as tumor or NK cells lacking CD28, suggests that it could have other unexpected roles.

We demonstrated here for the first time that ipilimumab can indeed exert direct effects on these cell populations, as it inhibits the growth of some tumor cell lines even in the absence of immune cells and efficiently activates the secretion of cytokines not only by T cells but also by NK cells. The comparable values of IFNγ secreted by the two populations of unfractionated hPBMCs and NK cells, stimulated in the presence of ipilimumab, shed light on the underestimated involvement of NK cells in immune responses modulated by ipilimumab. Furthermore, these findings open new scenarios on other possible mechanisms of action of this receptor, different from the above mentioned competition with CD28, and more likely involving its intracellular recruitment of phosphatases and/or signaling through PI3K, as it contains an YVKM motif able to bind to PI3K.

Interestingly, the EGFR tyrosine kinase receptor, overexpressed in many different tumors and well known for its oncogenic functions in tumor cells, has been recently proposed as a key regulator also of immune cells, as it has been found able to inhibit CTLs through different mechanisms [27]. Furthermore, clinical trials have evaluated the combination of ipilimumab with the anti-EGFR TKI erlotinib in NSCLC [29], highlighting the need of more specific EGFR drugs with low frequencies of adverse side effects. Thus, we considered the option of co-targeting these two receptors by firstly testing the combinations of ipilimumab with the anti-EGFR aptamer CL4, in order to verify if they show additive effects on both tumor cells and immune cells. This aptamer could become a safe and effective drug as it exhibits exquisite specificity toward EGFR-expressing cancer cells as reported by our group [33,34,35,36] and others [37,45,46]. Further, we proved that it does not cause toxicity [36] and immune stimulation upon administration to mice [34]. We found that the combinatorial treatments increased both their immunostimulatory activity on lymphocytes and their antitumor efficacy more efficiently than single agents. These results confirm the unexplored roles of CTLA-4 on non-immune cells as well as that of EGFR in modulation of immune responses, and suggest a possible crosstalk between the two receptors, even though further studies are needed to clarify the intracellular pathways involved.

On the basis of the encouraging results obtained with the combined treatment, we decided to construct a new bispecific immunoconjugate, made up of ipilimumab covalently linked to CL4, by using an efficacious strategy previously set up in our laboratory and successfully used with other two aptamer-antibody conjugates [39].

We found that the novel immunoconjugate, named CL4-ipilimumab, retained the binding of both the parental moieties to their target cells and also acquired a higher avidity accordingly to the presence of two binding sites in the chimeric construct. More importantly, the novel conjugate was able to induce the activation of both hPBMCs and NK cells more efficiently than the parental mAb and to display a more potent cytotoxicity against tumor target cells by combining the anti-cancer activities of the two different drugs. Furthermore, the conjugation of the anti-EGFR aptamer with the anti-CTLA-4mAb allowed also for the efficient killing of cancer cells mediated by effector and NK lymphocytes in co-cultures of tumor cells with both hPBMCs or NK cells, thus strongly increasing the cytotoxicity of the two partners in the chimeric construct.

Moreover, the interesting biological properties shown by this novel bispecific conjugate confirm that our antibody-aptamer conjugation strategy, previously successfully used for two additional bispecific conjugates [39], can be expanded to a large panel of different antibodies and it has the potential to become a universal platform for all the desired antibodies and aptamers. Indeed, once conjugated these bispecific constructs could acquire optimal biological features for therapeutic applications such as increased specificity for tumor cells, crosslinking of immune and cancer cells and improved pharmacokinetic and pharmacodynamic properties due to the combined advantages of small-size aptamers for increasing tumor penetration with those of the antibody, which allows for a longer half-life in circulation.

However, further in vivo studies, not yet performed due to the low yields of purification of this immunoconjugate, should be done in the future to confirm this hypothesis, already supported by previous findings relative to the extended in vivo pharmacokinetics of an aptamer linked to a mAb in a monospecific specific aptamer-antibody [47] immunoconjugate.

## 4. Materials and Methods 

### 4.1. Cell Cultures

Growth conditions for SK-BR-3, MCF-7 cell lines and LNCaP cancer cell lines were previously reported [39]. H9c2 cardiomyoblasts were cultured in Dulbecco’s Modified Eagles Medium enriched with 1mM sodium pyruvate (Gibco, Life Technologies, Grand Island, NE, USA). All culture media were supplemented with 10% heat-inactivated fetal bovine serum (FBS, Sigma Aldrich, St. Louis, MO, USA), 50 U/mL penicillin, 50 μg/mL streptomycin, and 1% of L-glutammine. All the cell lines, derived from ATCC (Gaithersburg, MD, USA), have been grown in humidified atmosphere containing 5% CO₂ at 37 °C. 

### 4.2. Isolation of Human Peripheral Blood Mononuclear Cells

Human PBMCs, isolated and frozen as previously reported [1,48], were thawed out and used after resting at 37 °C. 

### 4.3. Isolation of NK Cells

Human NK cells were isolated from hPBMCs by using the NK cell isolation kit (MACS, Miltenyi Biotec, Bergisch Gladbach, Germany) following the manufacturer’s guidelines. Briefly, the sample of hPBMCs was incubated with NK Cell Biotin-Antibody Cocktail for five minutes at 4 °C. NK cell microbead cocktail was then added and incubated for 10 min at 4 °C, diluted with buffer, and placed on a magnetic separator. The cells in the flow-through, corresponding to the enriched fraction of NK cells, were collected by centrifugation at 1200 rpm for eight minutes at 25 °C. The cell pellet was then resuspended in R10 medium and counted by using a Muse cell analyzer (Merck Millipore, 0500–3115, Darmdstadt, Germany).

### 4.4. Antibodies

The following antibodies were used: anti-CTLA4 mAb ipilimumab (Yervoy, Bristol Myers Squibb, NY, USA); anti-EGFR polyclonal antibody, anti-phospho44/42 MAPK (indicated as pERK) polyclonal antibody (Cell Signaling Technology, Danvers, MA, USA); anti-human IgG (H+L) HRP conjugate antibody (Promega, Madison, WI, USA); anti-vinculin (Santa Cruz Biotechnology, Santa Cruz, CA), and anti-actin antibody (Sigma-Aldrich).The following recombinant protein was used: Streptavidin-HRP (BioRad, Hercules, CA, USA).

### 4.5. Aptamers

2’Fluoro-pyrimidines (2’F-Py)-containing RNA sequences (CL4 and CL4Sc) were previously reported [33]. Unlabeled aptamers and the FAM-labeled, C6-NH₂-terminated and biotinylated derivatives were obtained from TriLink Biotechnologies (San Diego, CA, USA) and handled as reported [33,36,39].

### 4.6. Antibody–Oligonucleotide Conjugation

The aptamer-antibody conjugate was constructed by using an antibody–oligonucleotide solulink conjugation kit (TriLink Biotechnologies, San Diego, CA, USA), as previously reported [39]. Briefly, the amino terminated CL4 aptamer was labeled with the aromatic formylbenzamide group and covalently linked to hydrazinonicotamide-functionalized ipilimumab in the presence of the aniline reaction catalyst. The resulting aptamer-antibody immunoconjugate was purified on magnetic affinity matrix and eluted following the manufacturer’s recommendations.

### 4.7. Western Blotting Analysis of Cell Extracts

Western blotting analyses were carried out on cell extracts, prepared as previously reported [39], by incubating the nitrocellulose filters with indicated commercial primary antibodies followed by HRP-conjugated secondary antibody. Immunoreactive proteins were visualized as previously described [49]. 

### 4.8. ELISA Assays

Cell ELISA assays were carried out to measure the binding of the mAbs to the receptors exposed on the surface of tumor cells (2 × 10^5^ cells/well) and of human lymphocytes (4 × 10^5^ cells/well) previously activated with anti-CD3/CD28 beads or with SEB, by using NuncTM round-bottom 96-well plates. After blocking with PBS/BSA 6% for 20 min at RT, the intact cells were incubated in the absence or in the presence of biotinylated CL4 aptamer, ipilimumab or ipilimumab-CL4 immunoconjugate in PBS/BSA 3% buffer solution for 75 min at RT. After extensive washes with PBS the plates were incubated with HRP-conjugated streptavidin (to detect the binding of CL4 aptamer) for 30 min at RT or with an anti-human IgG (H+L) HRP conjugated antibody (to detect the binding of ipilimumab-CL4 immunoconjugate) for 1 h at RT. 

The calibration curve used for the quantification of CTLA-4 was obtained in a parallel sandwich ELISA assay performed by coating the plates with the commercial anti-CTLA-4 antibody (R and D Systems, Minneapolis, MN, USA) and incubating the purified CTLA-4 recombinant protein (R and D Systems) at increasing concentrations ranging from 0.2 to 50 ng/mL for 75 min at RT. After washes, the detection was carried out by an incubation with 200 nM biotinylated ipilimumab, followed by HRP-conjugated Streptavidin. The plates were then washed with PBS 1× and TMB substrate was used as previously reported [50].

### 4.9. Confocal Microscopy

SK-BR-3 and MCF-7 cells (10^5^ cells/well in 24-well), previously seeded on a coverslip for 24 h, were incubated at RT for 10 min with 200 nM FAM-labeled CL4, or for one hour with 200 nM ipilimumab or CL4-ipilimumab conjugate in BlockAid™ blocking solution (Life Technologies, Van Allen Way Carlsbad, CA USA). Then, cells were washed in PBS and fixed in 4% paraformaldehyde/PBS for 20 min. For the fluorescence visualization of ipilimumab and CL4-ipilimumab conjugate, non-permeabilized cells were incubated with FITC-labeled anti-human IgG antibody as previously reported [39].

### 4.10. Cell Growth Inhibition and Cytotoxicity Assays

To test the effects of ipilimumab, CL4, their combinatorial treatments, or CL4-ipilimumab on cell growth, tumor cells were plated in 96-well flat bottom plates: SK-BR-3 cells at the density of 1.5 × 10⁴ cells/well, LNCaP cells at the density of 7.5 × 10^3^ cells/well, MCF7 at the density of 1 × 10^3^, H9c2 at the density of 5 × 10^3^ and incubated for 16 h at 37 °C. CL4 aptamer (50 nM), the parental ipilimumab mAb (50 nM), their combination, or CL4-ipilimumab were added in the complete culture medium and incubated for 72 h. Cell counts were measured by the trypan blue exclusion test.

To test the effects of combinatorial treatments or those of the aptamer-antibody conjugate on co-cultures of tumor cells with hPBMCs or NK cells, SK-BR-3 cells (1.5 × 10^4^ cells/well) were plated in 96-well flat-bottom plates for 16 h. Then, lymphocytes were added (Effector: Target cell ratio 3:1) in the absence or presence of CL4 aptamer (30–200 nM), ipilimumab mAb (30–50 nM), used alone or in combination with CL4, or the immunoconjugate (30–50 nM) and incubated for 24 h at 37 °C. Controls included target cells incubated in the presence of effector cells, untreated or treated with CL4Sc (30 nM) or unrelated IgG mAb (30 nM). After the treatment, lymphocytes were removed, the supernatants were collected by centrifugation at 1200 rpm for 10 min, and the cells were washed and counted by the trypan blue exclusion test. Cell survival was expressed as percent of viable cells in the presence of the drugs with respect to the control cells untreated or treated with CL4 scrambled oligonucleotide sequence aptamer, used as a negative control.

### 4.11. Determination of Tumor Cell Lysis

Tumor cell lysis was defined by measuring the release of LDH, in the supernatant of cellular co-cultures, with a specific LDH detection kit (Thermofisher Scientific, Meridian Rd., Rockoford, IL, USA), according to the producer’s guidelines. Cell lysis was quantified by measuring the fold increase of LDH in treated cells compared to that present in the supernatant of untreated cells, used as a negative control. Cytolysis values were calculated by considering three independent experiments in which triplicate counts were determined.

### 4.12. Effects of the Antibodies and Immunoconjugate on Cytokine Release by Immune Cells

To verify the capacity of ipilimumab and the immunoconjugate to induce cytokine secretion, hPBMCs, or isolated NK cells were treated with SEB for 66 h. CD3⁺ T cells in unfractioned hPBMCs were activated with α-CD3/CD28 beads for 48 h. For each treatment CL4 aptamer, parental ipilimumab mAb, ipilimumab combined with CL4 aptamer or ipilimumab-CL4 immunoconjugate were added at concentrations ranging from 0.5 to 10 nM. Untreated or stimulated hPBMCs or NK cells, at the same concentrations, in the absence or in the presence of CL4Sc or unrelated IgG mAb were used as negative controls. 

To test the effects on co-cultures of SK-BR-3 cells with lymphocytes (effector:target ratio: 3:1), the cells were incubated for 24 h in the absence or in the presence of the immunoconjugate (30–50 nM), the parental ipilimumab mAb (30–50 nM) or CL4 aptamer (30–50 nM), used alone or in combination. Untreated or stimulated hPBMCs in the absence or in the presence of scrambled oligonucleotide sequence or unrelated IgG mAb were used as negative controls.

The concentration of IL-2 or IFNγ cytokines secreted in cell culture supernatant was measured by ELISA assays (DuoSet ELISA, R&D Systems, Minneapolis, MN, USA), according to the producer’s recommendations. Concentration values were reported as the mean of at least three determinations.

### 4.13. Statistical Analyses

Error bars were calculated by considering the results obtained by at least three independent experiments. Statistical analyses were assessed by Student’s t-test (two variables) or one-way ANOVA followed by Tukey’s multiple comparison test (more than two variables). Statistical significance was established as *** *p* ≤ 0.001; ** *p* <0.01; * *p* < 0.05. 

## 5. Conclusions

We investigated on the unexplored roles of CTLA-4 in tumor and NK cells, and on that of EGFR in the modulation of immune responses by testing new combinatorial treatments of ipilimumab with an anti-EGFR aptamer endowed with anti-tumor activity, and by generating for the first time a novel bispecific immunoconjugate, made up of these two compounds. 

The novel conjugate induces the activation of lymphocytes, including NK cells, more efficiently than the single agent treatments and combines the anti-tumor activities of these two different targeting agents, by efficiently activating the effector and NK lymphocytes against cancer cells.

## Supplementary Materials

The following are available online at https://www.mdpi.com/2072-6694/12/2/331/s1, Figure S1: Full length blot of Figure 1B, Figure S2: Effects of CL4 aptamer and Ipilimumab mAb on tumor cell intracellular signaling.
