# Supplementary material for: Ipilimumab and Its Derived EGFR Aptamer-Based Conjugate Induce Efficient NK Cell Activation against Cancer Cells
Source: Cancers (Basel). 2020 Feb 1;12(2):331. doi: 10.3390/cancers12020331 (PMC7072174; doi:10.3390/cancers12020331)
Supplement: Supplementary file 1 [file cancers-12-00331-s001.pdf]

Supplementary Materials

Ipilimumab and Its Derived Conjugate Induce Efficient NK Cell Activation Against Cancer Cells

Margherita Passariello, Simona Camorani, Cinzia Vetrei, Stefania Ricci, Laura Cerchia and Claudia De Lorenzo

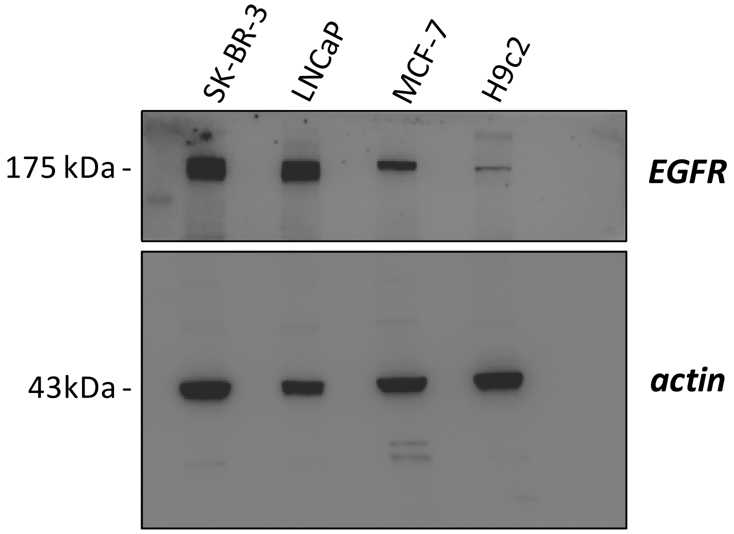

Figure S1. Full length blot of Figure 1B. The upper part of the filter was stained with an anti-EGFR polyclonal antibody (Cell Signaling Technology); the lower part of the same filter was stained with the anti-actin polyclonal antibody (SIGMA Aldrich). The intensity of the bands was normalized to actin by calculating the ratio of EGFR/actin signal intensities for each cell extract that was found to be 4.5 for SK-BR-3, 5.2 for LNCaP, 0.7 for MCF-7 and 0.07 for H9C2, respectively.

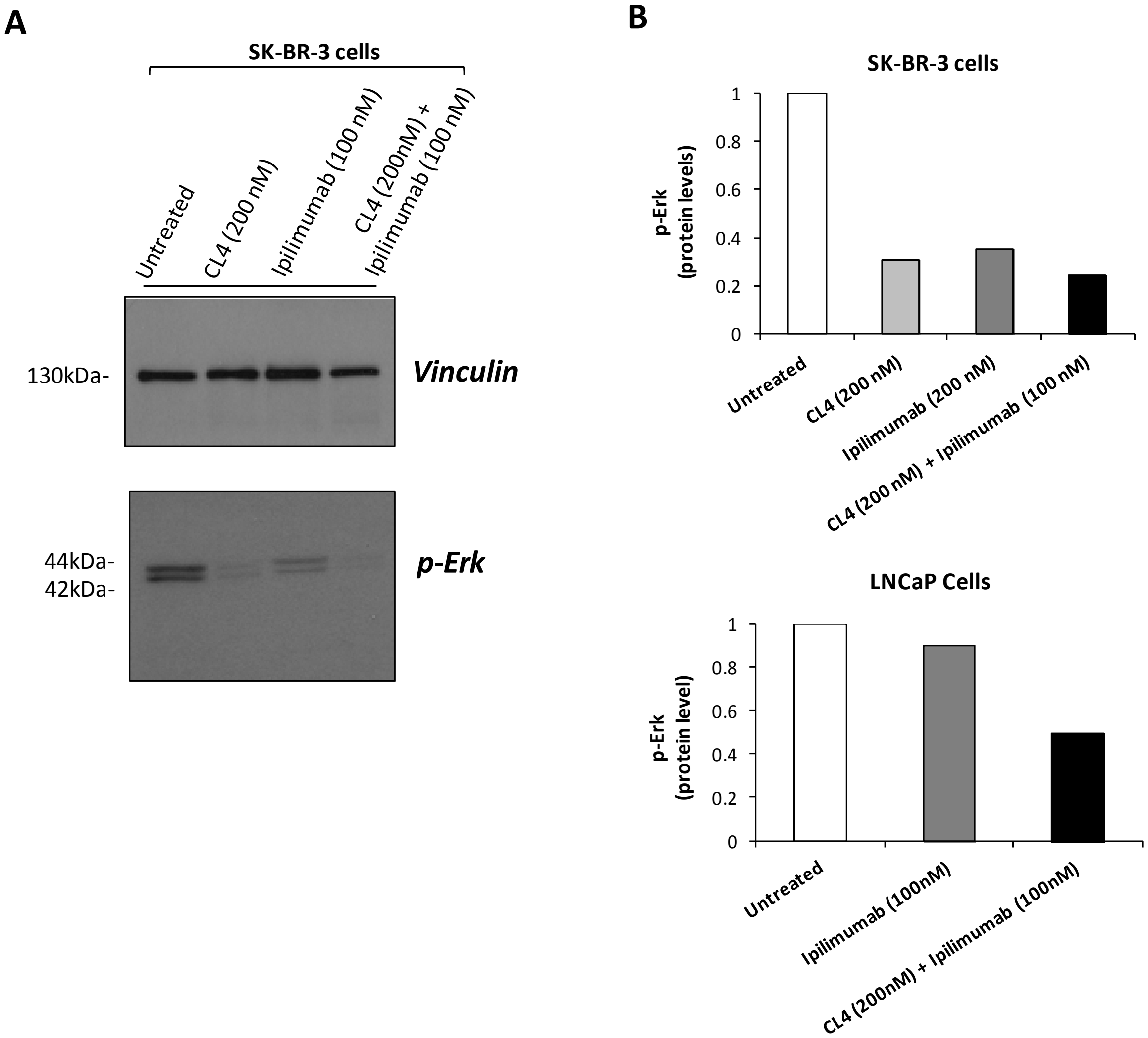

Figure S2. Effects of CL4 aptamer and Ipilimumab mAb on tumor cell intracellular signaling. (**A**) Western blotting analyses of extracts from SK-BR-3 or LNCaP tumor cells treated as indicated for 72 hours. Full length blots stained with anti-p-Erk or anti-Vinculin polyclonal antibodies. (**B**) Protein levels are expressed as fold increase of the band intensities with respect to those of untreated cells and normalized to vinculin for SK-BR-3 or actin for LNCaP cells.
